# *Announcement*: Sleep Awareness Week, April 23–29, 2017

**DOI:** 10.15585/mmwr.mm6615a6

**Published:** 2017-04-21

Sleep Awareness Week, the National Sleep Foundation’s annual campaign to educate the public about the importance of sleep in health and safety, will be observed April 23–29, 2017. The amount of sleep a person needs changes with age. Adults need ≥7 hours each night to promote optimal health and well-being (*1*); children and adolescents require even more sleep. Sleep needs decrease from 12–16 hours of sleep per 24 hours (including naps) for infants aged 4–12 months to 8–10 hours of sleep for teenagers aged 13–18 years (*2*). Children who regularly sleep less than the recommended amount are more likely to have behavior and learning problems, physical and mental health conditions such as obesity, diabetes, depression, or injuries (*2*). A regular bedtime routine can help children get adequate sleep. The American Academy of Pediatrics provides advice for parents at https://www.healthychildren.org/English/healthy-living/sleep/. Additional details about how much sleep is recommended across a lifespan is available at https://www.cdc.gov/sleep/about_sleep/how_much_sleep.html.
